# Sex Differences in the Associations among Insulin Resistance Indexes with Metabolic Syndrome: A Large Cross-Sectional Study

**DOI:** 10.1155/2024/3352531

**Published:** 2024-09-30

**Authors:** Wenkang Zhang, Chen Chen, Mingkang Li, Gaoliang Yan, Chengchun Tang

**Affiliations:** ^1^ School of Medicine Southeast University, Nanjing, Jiangsu, China; ^2^ Department of Cardiology Zhongda Hospital Southeast University, Nanjing, Jiangsu, China; ^3^ School of Cyber Science and Engineering Southeast University, Nanjing, Jiangsu, China

## Abstract

**Purpose:**

Metabolic syndrome (MetS) is closely related to insulin resistance (IR), and the sex differences have not been fully explored. This study was aimed to investigate the sex differences in the associations among IR indexes with MetS in a large population.

**Methods:**

A total of 60,799 participants were enrolled in the current study. MetS was defined using the National Cholesterol Education Program Adult Treatment Panel III criteria. The following IR indexes were evaluated: triglyceride-glucose (TyG) index, TyG-waist circumference (TyG-WC), TyG-waist to height ratio (TyG-WHtR), TyG-body mass index (TyG-BMI), triglyceride to high-density lipoprotein cholesterol (TG/HDL-C), and metabolic score for IR (MetS-IR). Factors associated with MetS were examined using logistic regressions. The receiver operating characteristic curves were used to evaluate the predictive value of the IR indexes for MetS.

**Results:**

The prevalence of MetS was 11.8% (*n* = 4097) for males and 5.4% (*n* = 1390) for females and increased with age in both subgroups. The IR index levels of male patients were higher than female patients (all *p* < 0.001). The IR indexes were independent risk factors for MetS except for TyG-WHtR and TG/HDL-C in female patients. TyG had the greatest area under the curve (AUC) (AUC, 0.930; 95% CI, 0.928–0.933; *p* < 0.001) in the male patients and TyG-WHtR (AUC, 0.916; 95% CI, 0.913–0.920; *p* < 0.001) in the female patients. The AUCs of 6 IR indexes combination were 0.960 (95% CI, 0.957–0.962; *p* < 0.001) and 0.962 (95% CI, 0.959–0.964; *p* < 0.001), with the sensitivities of 91.29% and 90.94%, the specificities of 88.27% and 89.51% in male and female groups, respectively.

**Conclusions:**

The IR index levels are higher in male than female patients. In IR indexes, TyG has the highest AUC in male patients and TyG-WHtR in female patients. The combination of IR indexes improved diagnostic efficiency compared with a single parameter. Moreover, the IR indexes are independently associated with MetS except for TyG-WHtR and TG/HDL-C in female patients. Our findings indicate that the multi-index association of IR indexes may play a potential role in MetS diagnosis, and understanding the sex differences in risk factors for MetS may help doctors properly implement more individualized prevention strategies.

## 1. Introduction

Metabolic syndrome (MetS) is defined as a cluster of risk factors, comprising abdominal obesity, hyperglycemia, dyslipidemia, and elevated blood pressure (BP), for atherosclerotic cardiovascular diseases, diabetes mellitus, and vascular and neurological complications [1, 2]. A global prevalence of 12.5% to 31.4%, especially about 26% in Spain, makes MetS become a health issue of worldwide concern [3, 4].

The underlying etiologies of the MetS are overweight, physical inactivity, hereditary susceptibility, unhealthy diet, and metabolic dyslipidemia [1, 5–7]. Insulin resistance (IR) due to an accumulation of adipose tissue and tissue dysfunction is the critical point [8]. It refers to a reduced insulin function after binding with insulin receptors [9]. IR severity measurement requires insulin tests or invasive detection, which is inappropriate for large-scale epidemiological investigations [10]. Several non-insulin-based IR indicators as substitutes have been put forward, mainly including the triglyceride-glucose (TyG) index, TyG-waist circumference (TyG-WC), TyG-waist to height ratio (TyG-WHtR), TyG-body mass index (TyG-BMI), triglyceride to high-density lipoprotein cholesterol (TG/HDL-C), and metabolic score for IR (MetS-IR), which made IR measurement straightforward [11].

Although several studies have found a correlation between IR indexes and MetS, the sex differences have not been fully explored [11–13]. Therefore, our study was aimed to investigate the sex differences in the associations among IR indexes with MetS in a large population to provide more personalized strategies for the prevention and diagnosis of MetS.

## 2. Methods

### 2.1. Study Design and Populations

This research is a secondary analysis based on a cross-sectional study involving a working population in the Balearic Islands (Spain) from 2012 to 2016, which was aimed to confirm a noninvasive method using only two anthropometric variables, WHtR and BP, for the early detection of MetS in a large representative sample. 69,581 local employees were invited from different economic sectors aged 20 to 70 years old to participate in the study, of which 8782 declined the invitation, and the remaining 60,799 employees were included in the preliminary study. Participants were given written informed consent, and the study protocol complied with the Declaration of Helsinki. More details of the original study were described elsewhere [14].

### 2.2. Data Collection

The data were extracted from the DRYAD database (https://www.Datadryad.org/). According to the data sharing policy, all researchers are free to download the original data and use the data for secondary analysis without harming the rights and interests of the authors. Following the policy, we needed to cite data sources when using these data [15]. The original data variables we required in this study were age, smoking, systolic BP (SBP), diastolic BP (DBP), BMI, percentage of body fat (%BF), a body shape index (ABSI), WC, WHtR, cholesterol, TG, HDL-C, low-density lipoprotein cholesterol (LDL-C), and glucose. The detailed data collection and measurement methods were mentioned in the original research by Manuel Romero-Saldaña et al. [14].

### 2.3. Definitions

In the original study, BMI was calculated as body weight (kg) divided by height (m) squared. %BF was calculated according to the Deurenberg equation: %BF = 1.2 × BMI (kg/m^2^) + 0.23 × age (years) − 10.8 × gender (female = 0, male = 1)–5.4. ABSI was calculated as WC (cm)/[BMI (kg/m^2^)]^2/3^ × [height (m)]^1/2^. WHtR was calculated as WC (cm) divided by height (cm) [14].

In the present study, the formulas for calculating the IR indexes were as follows: TyG = Ln [fasting TG (mg/dL) × fasting glucose (mg/dL)/2], TyG-BMI = TyG × BMI (kg/m^2^), TyG-WC = TyG × WC (cm), TyG-WHtR = TyG × WHtR, TG/HDL-C = fasting TG (mg/dL)/fasting HDL-C (mg/dL), and MetS-IR = Ln [2 × fasting glucose (mg/dL) + fasting TG (mg/dL)] × BMI (kg/m^2^)/Ln [fasting HDL-C (mg/dL)] [11].

MetS was defined according to the National Cholesterol Education Program Adult Treatment Panel III (NCEP-ATP III) criteria when at least three of the following five clinical features are present: (i) abdominal obesity (WC ≥ 102 cm in males and WC ≥ 88 cm in females); (ii) triglycerides ≥150 mg/dL; (iii) HDL-C < 40 mg/dL in males and <50 mg/dL in females; (iv) BP ≥ 130/85 mmHg; and (v) fasting glucose ≥100 mg/dL [16]. In our study, the study populations were stratified by sex and whether they suffered from MetS to explore the differences in IR indexes between male and female MetS patients and between male or female MetS patients and normal controls.

### 2.4. Statistical Analysis

All data in this study were processed by SPSS version 25.0 (SPSS Inc., Chicago, IL, USA) and MedCalc version 20.0 (Med-Calc Software, Mariakerke, Belgium). Continuous variables were summarized using median (IQR) and categorical variables using frequencies and percentages. Differences in continuous variables were compared by the Mann–Whitney *U*-test and categorical variables by a chi-square test or Fisher's exact test. The correlations between the IR indexes were performed by Spearman's correlation test. Factors associated with MetS were examined using univariate and multivariate logistic regressions. Variables with statistical differences in univariate analysis were adjusted as confounders in multivariate logistic regression. The receiver operating characteristic (ROC) curves were used to evaluate the predictive values of the IR indexes for MetS. Two-sided *p* values of <0.05 were considered statistically significant.

## 3. Results

### 3.1. Study Populations and Prevalence of MetS

**Figure 1:**
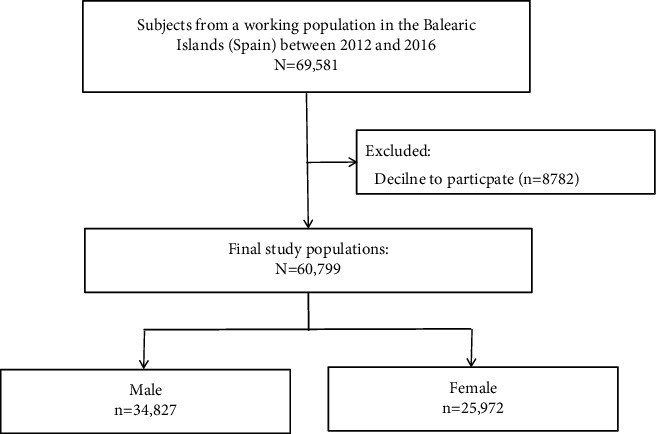
Flow chart of the study populations.

**Figure 2:**
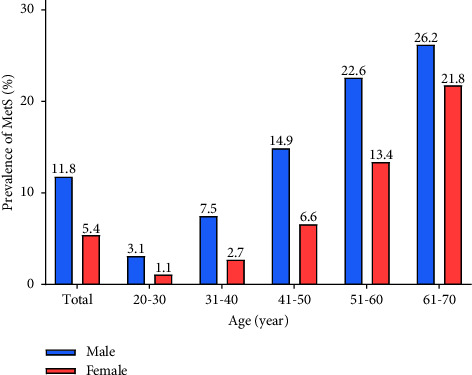
Prevalence of MetS by gender according to the age group. MetS: metabolic syndrome.

The flow chart of the study populations is shown in Figure 1. After excluding 8782 subjects unwilling to participate, final 60,799 individuals were enrolled in the study, including 34,827 (57.3%) males and 25,972 (42.7%) females. The prevalence of MetS was 11.8% (*n* = 4097) for males and 5.4% (*n* = 1390) for females and increased with age in both subgroups (Figure 2).

### 3.2. Baseline Characteristics of the Study Populations

Table 1 exhibits the baseline clinical characteristics of the study populations stratified by sex and MetS or not. Compared with the male group without MetS, the male MetS patients were older, had higher levels of SBP, DBP, BMI, %BF, ABSI, WC, WHtR, cholesterol, TG, LDL-C, and glucose, and lower levels of HDL-C (all *p* < 0.001). Additionally, the smokers tended to suffer from MetS (*p* < 0.001). Likewise, similar results were found in the female patients. Compared with the male MetS patients, the female patients were older and had less proportion of smokers, lower levels of SBP, ABSI, WC, WhtR, TG, LDL-C, and glucose, and higher levels of BMI, %BF, and HDL-C (all *p* < 0.05).

### 3.3. Comparisons and Correlations of the IR Indexes of Different Genders

**Figure 3:**
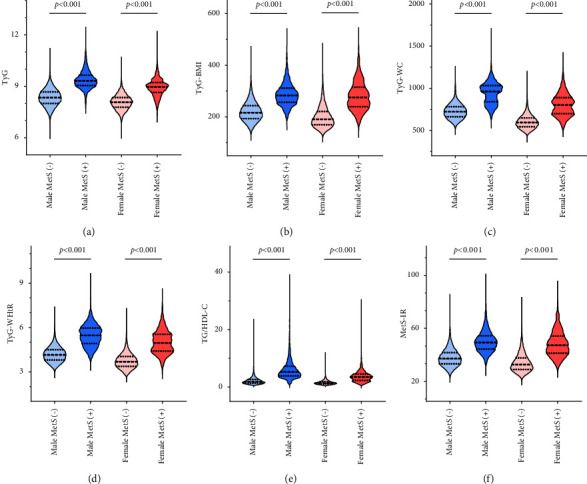
Comparisons of IR indexes by gender. IR: insulin resistance; MetS: metabolic syndrome; TyG: triglyceride-glucose; BMI: body mass index; WC: waist circumference; WHtR: waist-to-height ratio; TG: triglyceride; HDL-C: high-density lipoprotein cholesterol; MetS-IR: metabolic score for insulin resistance.

From Figure 3, we can see that both male and female MetS patients had higher TyG [male: 9.3 (9,0, 9.7) vs. 8.3 (8.0, 8.7), *p* < 0.001; female: 9.0 (8.6, 9.2) vs. 8.1 (7.8, 8.3), *p* < 0.001], TyG-BMI [male: 282.9 (256.8, 311.2) vs. 216.5 (193.6, 243.2), *p* < 0.001; female: 275.3 (239.7, 314.7) vs. 191.2 (169.8, 220.9), *p* < 0.001], TyG-WC [male: 960.5 (839.4, 1030.9) vs. 720.8 (662.0, 782.1), *p* < 0.001; female: 800.0 (698.2, 887.5) vs. 592.7 (543.5, 648.1), *p* < 0.001], TyG-WHtR [male: 5.5 (4.9, 6.0) vs. 4.2 (3.8, 4.5), *p* < 0.001; female: 5.0 (4.4, 5.5) vs. 3.7 (3.4, 4.0), *p* < 0.001], TG/HDL-C [male: 5.2 (3.9, 7.2) vs. 1.9 (1.3, 2.6), *p* < 0.001; female: 3.5 (2.3, 4.5) vs. 1.4 (1.1, 1.9), *p* < 0.001], and MetS-IR [male: 49.4 (44.5, 54.5) vs. 37.2 (33.2, 41.7), *p* < 0.001; female: 47.3 (41.3, 54.2) vs. 32.7 (29.1, 37.8), *p* < 0.001] compared with those without MetS. Moreover, the levels of TyG, TyG-BMI, TyG-WC, TyG-WHtR, TG/HDL-C, and MetS-IR were higher in male than female MetS patients (all *p* < 0.001). Spearman correlation analyses showed that the IR indexes were correlated positively with each other (all *p* < 0.001) in male and female patients (Tables 2 and 3).

### 3.4. The Associations of the IR Indexes with MetS

The results of univariate logistic regression analyses are shown in Table 4. Age, smoking, SBP, DBP, BMI, %BF, ABSI (≥0.073), WC, WHtR (≥0.492), cholesterol, TG, HDL-C, LDL-C, glucose, TyG (≥8.3), TyG-BMI, TyG-WC, TyG-WHtR, TG/HDL-C, and MetS-IR were significantly associated with MetS in both male and female subgroups (all *p* < 0.05).  Table 5 describes the results of the multivariate logistic regression analyses. After adjusting for age, smoking, SBP, DBP, BMI, %BF, ABSI (≥0.073), WC, WHtR (≥0.492), cholesterol, TG, HDL-C, LDL-C, and glucose, all the IR indexes, including TyG (≥8.3) [odds ratio (OR), 2.363; 95% CI, 1.842–3.060; *p* < 0.001], TyG-BMI (OR, 1.198; 95% CI, 1.182–1.215; *p* < 0.001), TyG-WC (OR, 1.061; 95% CI, 1.056–1.065; *p* < 0.001), TyG-WHtR (OR, 5.557; 95% CI, 4.194–7.363; *p* < 0.001), TG/HDL-C (OR, 1.523; 95% CI, 1.315–1.765; *p* < 0.001), and MetS-IR (OR, 2.903; 95% CI, 2.660–3.168; *p* < 0.001), were independent risk factors of male MetS patients, while only TyG (≥8.3) (OR, 1.381; 95% CI, 1.081–1.765; *p*=0.010), TyG-BMI (OR, 1.060; 95% CI, 1.041–1.080; *p* < 0.001), TyG-WC (OR, 1.025; 95% CI, 1.018–1.032; *p* < 0.001), and MetS-IR (OR, 2.210; 95% CI, 1.921–2.543; *p* < 0.001) were independent risk factors of female MetS patients, except for TyG-WHtR and TG/HDL-C (all *p* > 0.05).

### 3.5. Diagnostic Values of the IR Indexes by ROC Curves on MetS

**Figure 4:**
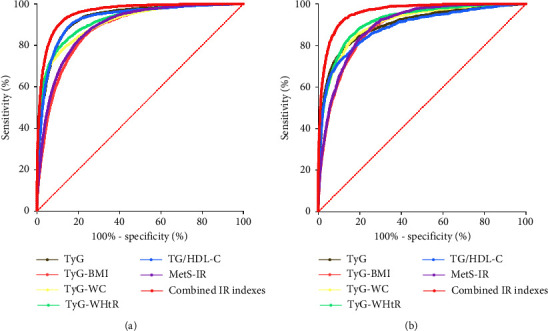
The ROC curve analyses for the IR indexes among (a) males and (b) females. ROC: receiver operating characteristic; IR: insulin resistance; TyG: triglyceride-glucose; BMI: body mass index; WC: waist circumference; WHtR: waist-to-height ratio; TG: triglyceride; HDL-C: high-density lipoprotein cholesterol; MetS-IR: metabolic score for insulin resistance.

On ROC curve analyses (Figure 4), among all the IR indexes, TyG had the greatest area under the curve (AUC) (AUC, 0.930; 95% CI, 0.928–0.933; *p* < 0.001) in the male patients and TyG-WHtR (AUC, 0.916; 95% CI, 0.913–0.920; *p* < 0.001) in the female patients. Furthermore, the combination of 6 IR indexes elevated the AUCs to 0.960 and 0.962, the sensitivities to 91.29% and 90.94%, and the specificities to 88.27% and 89.51% in male and female groups, respectively, which indicated that the multi-index association was a precise predictor for MetS. Separate ROC analysis results of AUCs, cut-off values, sensitivities, specificities, and Youden indexes of the IR indexes for MetS in the males and females are exhibited in Tables 6 and 7.

## 4. Discussion

In this study, we uncovered the gender-based differences in risk factors, levels, and diagnostic values of the IR indexes for MetS. Our research is the first large-scale study on the sex differences in the associations among the IR indexes with MetS.

According to a meta-analysis of global data from 28 million individuals, the global prevalence of MetS was increased with age and was higher in women than men [3]. Our study also revealed that the older the age, the higher the prevalence. However, the male MetS patients were more than females. The reason may be the participants' selection and regional differences.

IR plays a crucial role in MetS. Both visceral and subcutaneous fats promote IR, and the former is predominant. A higher lipolysis rate in visceral fat contributes to a higher free fatty acid level and the following fat accumulation in the liver, which conversely induces IR. Moreover, adipocytokines released by visceral fat may also induce IR [17]. These can explain why the IR indexes were higher in male and female MetS patients and were associated with MetS.

TyG has been testified to be correlated with direct IR markers and was extensively studied in MetS [18, 19]. A systematic review and meta-analysis revealed that the pooled diagnostic OR for MetS was 16.58, and the combined AUC was 0.87, with sensitivity and specificity of 80% and 81%, respectively [19]. Our study also found that TyG (≥8.3) was the independent risk factor for MetS, and the TyG index was a valuable index for MetS screening.

MetS is strongly related to lipid abnormalities [20]. The lipid-related indicators, such as TG/HDL-C, total cholesterol (TC)/HDL-C, and LDL-C/HDL-C, have been confirmed as risk factors for MetS and alternatives for MetS diagnosis in both genders. Among them, the TG/HDL-C was the best ratio to discriminate individuals with and without MetS and has a greater correlation with the IR [21, 22]. Interestingly, TG/HDL-C was not an independent risk factor of female MetS patients in our study. The reason might be that it was strongly correlated with other risk factors.

In 2018, Bello-Chavolla OY et al. proposed a new non-insulin-based MetS‐IR for the first time. Scanty evidence manifested that METS‐IR was related to diabetes mellitus, hypertension, and MetS [11, 23, 24]. Mirr et al. found that compared with other IR indicators, METS‐IR did not have a better diagnostic value for MetS in men and women [11], which was consistent with our study. We also found that MetS-IR was an independent risk factor for male and female MetS patients.

In a Nigeria study, TyG-WHtR had the greatest AUC for MetS diagnosis in all participants, followed by TyG-WC, TyG-BMI, TyG, and WHtR. Gender subgroup analysis found that TyG-WC had the largest AUC in both genders [13]. A Poland study showed that TyG and TG/HDL-C ranked first and second for the highest AUC in all subjects and gender subgroups. However, when subjects were stratified by proper BMI, overweight, or obesity, the IR index with the highest AUC differed in gender subgroups [11]. Although these studies were conducted in different populations and areas, no uniform diagnostic index was identified. The current study found that TyG has the highest AUC in male patients and TyG-WHtR in female patients. What is more, the combination of the TyG index, TyG-WC, TyG-WHtR, TyG-BMI, TG/HDL-C, and MetS-IR together can achieve extremely high AUC and high sensitivity and specificity in comparison with only a single parameter, no matter in male and female subgroups, suggesting that the combined IR indexes have the best discriminating power to identify MetS.

Limitations of this study must be considered. First, this study was carried out in a specific region, so the findings may not be extrapolated. Second, the MetS diagnosis was according to the NCEP-ATP III definition, and no more diagnostic criteria were used for comparison. Third, some crucial factors affecting MetS, such as exercise, economic status, medication and circadian disruption, were absent. Fourth, this study failed to compare IR indexes with insulin-based IR markers, such as the homeostasis model assessment of IR (HOMA-IR) and the quantitative insulin-sensitivity check index (QUICKI), in MetS diagnosis. Finally, the nature of cross-sectional research denied the possibility of examining the cause-and-effect relationship because disease and factors coexist.

## 5. Conclusions

In conclusion, the IR index levels are higher in male than female patients. In IR indexes, TyG has the highest AUC in male patients and TyG-WHtR in female patients. The combination of IR indexes improved diagnostic efficiency compared with a single parameter. Moreover, the IR indexes are independently associated with MetS except for TyG-WHtR and TG/HDL-C in female patients. Our findings indicate that the multi-index association of IR indexes may play a potential role in MetS diagnosis, and understanding the sex differences in risk factors for MetS may help doctors properly implement more individualized prevention strategies.

## Figures and Tables

**Table 1:** Baseline characteristics of the study populations.

Variables	Male (*n* = 34,827)			Female (*n* = 25,972)		
	MetS (−) (*n* = 30,730)	MetS (+) (*n* = 4097)	*p*	MetS (−) (*n* = 24,582)	MetS (+) (*n* = 1390)	*p*
Age (years)	39.0 (32.0, 47.0)	47.0 (40.0, 54.0)	<0.001		38.3 (31.0, 46.0)	<0.001
Smoking (*n*, %)	11018 (35.9)	1728 (42.2)	<0.001	8023 (32.6)	408 (29.4)	0.011
SBP (mmHg)	120.0 (114.0, 130.0)	135.0 (130.0, 146.0)	<0.001	110.0 (104.0, 120.0)	130.0 (120.0, 140.0)	<0.001
DBP (mmHg)	75.0 (70.0, 80.0)	80.0 (80.0, 90.0)	<0.001	70.0 (60.0, 78.0)	80.0 (75.0, 90.0)	<0.001
BMI (kg/m^2^)	25.9 (23.8, 28.4)	30.1 (27.5, 33.1)	<0.001	23.7 (21.4, 27.0)	30.7 (27.2, 34.8)	<0.001
%BF	24.2 (20.5, 28.2)	30.9 (27.5, 34.6)	<0.001	32.3 (28.5, 36.9)	42.5 (38.6, 47.4)	<0.001
ABSI	0.075 (0.071, 0.079)	0.079 (0.073, 0084)	<0.001	0.069 (0.065, 0.074)	0.071 (0.064, 0.079)	<0.001
WC (cm)	86.0 (81.0, 92.0)	104.0 (91.0, 108.0)	<0.001	74.0 (68.1, 79.0)	90.0 (78.0, 97.2)	<0.001
WHtR	0.497 (0.465, 0.531)	0.589 (0.536, 0.623)	<0.001	0.459 (0.424, 0.491)	0.562 (0.497, 0.609)	<0.001
Cholesterol (mg/dL)	192.0 (168.0, 216.0)	220.0 (195.0, 248.7)	<0.001	189.0 (166.0, 214.0)	219.0 (196.8, 247.0)	<0.001
TG (mg/dL)	96.0 (70.0, 129.0)	218.1 (168.0, 298.0)	<0.001	77.0 (59.0, 98.2)	162.3 (108.0, 204.0)	<0.001
HDL-C (mg/dL)	52.0 (47.0, 55.3)	42.0 (36.0, 50.0)	<0.001	55.0 (50.0, 60.0)	46.0 (43.0, 50.0)	<0.001
LDL-C (mg/dL)	119.6 (95.4, 143.8)	130.4 (101.2, 158.0)	<0.001	117.8 (93.0, 143.0)	140.2 (115.4, 166.2)	<0.001
Glucose (mg/dL)	87.0 (79.0, 95.0)	101.0 (89.0, 112.0)	<0.001	83.0 (77.0, 90.4)	100.0 (88.0, 110.0)	<0.001

Data are presented as *n* (%) or median (25th, 75th). MetS: metabolic syndrome; SBP: systolic blood pressure; DBP: diastolic blood pressure; BMI: body mass index; %BF: percentage of body fat; ABSI: a body shape index; WC: waist circumference; WHtR: waist-to-height ratio; TG: triglyceride; HDL-C: high-density lipoprotein cholesterol; LDL-C: low-density lipoprotein cholesterol.

**Table 2:** The correlations between the IR indexes in the male group.

Variables	TyG	TyG-BMI	TyG-WC	TyG-WHtR	TG/HDL-C	MetS-IR
TyG	1					
TyG-BMI	0.663^∗^	1				
TyG-WC	0.713^∗^	0.810^∗^	1			
TyG-WHtR	0.729^∗^	0.827^∗^	0.953^∗^	1		
TG/HDL-C	0.937^∗^	0.636^∗^	0.683^∗^	0.699^∗^	1	
MetS-IR	0.593^∗^	0.974^∗^	0.775^∗^	0.796^∗^	0.604^∗^	1

IR: insulin resistance; TyG: triglyceride-glucose; BMI: body mass index; WC: waist circumference; WHtR: waist-to-height ratio; TG: triglyceride; HDL-C: high-density lipoprotein cholesterol; MetS-IR: metabolic score for insulin resistance. ^∗^*p* < 0.001.

**Table 3:** The correlations between the IR indexes in the female group.

Variables	TyG	TyG-BMI	TyG-WC	TyG-WHtR	TG/HDL-C	MetS-IR
TyG	1					
TyG-BMI	0.561^∗^	1				
TyG-WC	0.579^∗^	0.739^∗^	1			
TyG-WHtR	0.590^∗^	0.772^∗^	0.956^∗^	1		
TG/HDL-C	0.891^∗^	0.535^∗^	0.524^∗^	0.540^∗^	1	
MetS-IR	0.465^∗^	0.970^∗^	0.690^∗^	0.727^∗^	0.508^∗^	1

IR: insulin resistance; TyG: triglyceride-glucose; BMI: body mass index; WC: waist circumference; WHtR: waist-to-height ratio; TG: triglyceride; HDL-C: high-density lipoprotein cholesterol; MetS-IR: metabolic score for insulin resistance. ^∗^*p* < 0.001.

**Table 4:** Factors associated with MetS from univariate logistic regression analyses.

Variables	Male			Female		
	OR	95% CI	*p*	OR	95% CI	*p*
Age	1.071	1.067–1.074	<0.001	1.095	1.089–1.102	<0.001
Smoking	1.305	1.221–1.394	<0.001	0.858	0.762–0.965	0.011
SBP	1.053	1.050–1.055	<0.001	1.066	1.063–1.069	<0.001
DBP	1.081	1.078–1.085	<0.001	1.104	1.098–1.109	<0.001
BMI	1.239	1.230–1.249	<0.001	1.218	1.206–1.229	<0.001
%BF	1.199	1.192–1.206	<0.001	1.194	1.185–1.204	<0.001
ABSI (≥0.073)	1.802	1.672–1.942	<0.001	2.045	1.833–2.281	<0.001
WC	1.155	1.150–1.160	<0.001	1.113	1.108–1.119	<0.001
WHtR (≥0.492)	7.190	6.488–7.967	<0.001	10.726	9.422-12.210	<0.001
Cholesterol	1.019	1.018–1.020	<0.001	1.021	1.019–1.022	<0.001
TG	1.019	1.019–1.020	<0.001	1.026	1.025–1.027	<0.001
HDL-C	0.849	0.845–0.854	<0.001	0.879	0.872–0.885	<0.001
LDL-C	1.007	1.006–1.008	<0.001	1.015	1.014–1.016	<0.001
Glucose	1.034	1.032–1.036	<0.001	1.055	1.052–1.059	<0.001
TyG (≥8.3)	44.731	35.858–55.800	<0.001	19.592	16.556–23.185	<0.001
TyG-BMI	1.035	1.034–1.036	<0.001	1.028	1.027–1.029	<0.001
TyG-WC	1.017	1.016–1.017	<0.001	1.015	1.014–1.016	<0.001
TyG-WHtR	19.115	17.701–20.642	<0.001	11.776	10.709–12.950	<0.001
TG/HDL-C	2.440	2.381–2.501	<0.001	3.558	3.379–3.747	<0.001
MetS-IR	1.232	1.225–1.240	<0.001	1.178	1.170–1.186	<0.001

MetS: metabolic syndrome; OR: odds ratio; CI: confidence interval; SBP: systolic blood pressure; DBP: diastolic blood pressure; BMI: body mass index; %BF: percentage of body fat; ABSI: a body shape index; WC: waist circumference; WHtR: waist-to-height ratio; TG: triglyceride; HDL-C: high-density lipoprotein cholesterol; LDL-C: low-density lipoprotein cholesterol; TyG: triglyceride-glucose; MetS-IR: metabolic score for insulin resistance.

**Table 5:** The multivariate logistic regression analyses in an adjusted model.

Variables	Male			Female		
	OR	95% CI	*p*	OR	95% CI	*p*
TyG (≥8.3)	2.363	1.842–3.060	<0.001	1.381	1.081–1.765	0.010
TyG-BMI	1.198	1.182–1.215	<0.001	1.060	1.041–1.080	<0.001
TyG-WC	1.061	1.056–1.065	<0.001	1.025	1.018–1.032	<0.001
TyG-WHtR	5.557	4.194–7.363	<0.001	1.389	0.889–2.169	0.149
TG/HDL-C	1.523	1.315–1.765	<0.001	0.924	0.634–1.346	0.679
MetS-IR	2.903	2.660–3.168	<0.001	2.210	1.921–2.543	<0.001

Model: adjusted for variables including age, smoking, SBP, DBP, BMI, %BF, ABSI (≥0.073), WC, WHtR (≥0.492), cholesterol, TG, HDL-C, LDL-C, and glucose. MetS: metabolic syndrome; OR: odds ratio; CI: confidence interval; TyG: triglyceride-glucose; BMI: body mass index; WC: waist circumference; WHtR: waist-to-height ratio; TG: triglyceride; HDL-C: high-density lipoprotein cholesterol; MetS-IR: metabolic score for insulin resistance.

**Table 6:** The ROC curve analyses for the IR indexes in the male group.

Variables	AUC (95% CI)	Cut-off value	Sensitivity (%)	Specificity (%)	Youden index
TyG	0.930 (0.928–0.933)	8.9	87.89	85.55	0.73
TyG-BMI	0.880 (0.877–0.883)	246.1	84.45	77.13	0.62
TyG-WC	0.911 (0.908–0.914)	826.9	77.89	87.84	0.66
TyG-WHtR	0.920 (0.917–0.923)	4.8	80.55	87.80	0.68
TG/HDL-C	0.928 (0.925–0.931)	3.1	88.85	84.94	0.74
MetS-IR	0.890 (0.887–0.894)	42.8	83.21	79.39	0.63
Combined IR indexes	0.960 (0.957–0.962)	—	91.29	88.27	0.80

ROC: receiver operating characteristic; IR: insulin resistance; AUC: area under the curve; TyG: triglyceride-glucose; BMI: body mass index; WC: waist circumference; WHtR: waist-to-height ratio; TG: triglyceride; HDL-C: high-density lipoprotein cholesterol; MetS-IR: metabolic score for insulin resistance.

**Table 7:** The ROC curve analyses for the IR indexes in the female group.

Variables	AUC (95% CI)	Cut-off value	Sensitivity (%)	Specificity (%)	Youden index
TyG	0.900 (0.897–0.904)	8.5	80.29	85.96	0.66
TyG-BMI	0.889 (0.885–0.893)	223.0	86.69	76.24	0.63
TyG-WC	0.907 (0.903–0.911)	668.5	85.04	81.78	0.67
TyG-WHtR	0.916 (0.913–0.920)	4.2	84.68	84.44	0.69
TG/HDL-C	0.886 (0.883–0.890)	2.4	74.46	87.89	0.62
MetS-IR	0.895 (0.891–0.899)	38.9	84.96	78.95	0.64
Combined IR indexes	0.962 (0.959–0.964)	—	90.94	89.51	0.81

ROC: receiver operating characteristic; IR: insulin resistance; AUC: area under the curve; TyG: triglyceride-glucose; BMI: body mass index; WC: waist circumference; WHtR: waist-to-height ratio; TG: triglyceride; HDL-C: high-density lipoprotein cholesterol; MetS-IR: metabolic score for insulin resistance.

## Data Availability

The datasets analyzed during the current study are available in the DRYAD repository, https://doi.org/10.5061/dryad.cb51t54 [15].
